# Cancer Chemoprevention and Piperine: Molecular Mechanisms and Therapeutic Opportunities

**DOI:** 10.3389/fcell.2018.00010

**Published:** 2018-02-15

**Authors:** Rafiq A. Rather, Madhulika Bhagat

**Affiliations:** School of Biotechnology, University of Jammu, Jammu, India

**Keywords:** cancer, chemoprevention, piperine, apoptosis, cell cycle arrest, self-renewal, bioavailability

## Abstract

Cancer is a genetic disease characterized by unregulated growth and dissemination of malignantly transformed neoplastic cells. The process of cancer development goes through several stages of biochemical and genetic alterations in a target cell. Several dietary alkaloids have been found to inhibit the molecular events and signaling pathways associated with various stages of cancer development and therefore are useful in cancer chemoprevention. Cancer chemoprevention has long been recognized as an important prophylactic strategy to reduce the burden of cancer on health care system. Cancer chemoprevention assumes the use of one or more pharmacologically active agents to block, suppress, prevent, or reverse the development of invasive cancer. Piperine is an active alkaloid with an excellent spectrum of therapeutic activities such as anti-oxidant, anti-inflammatory, immunomodulatory, anti-asthmatic, anti-convulsant, anti-mutagenic, antimycobacterial, anti-amoebic, and anti-cancer activities. In this article, we made an attempt to sum up the current knowledge on piperine that supports the chemopreventive potential of this dietary phytochemical. Many mechanisms have been purported to understand the chemopreventive action of piperine. Piperine has been reported to inhibit the proliferation and survival of many types of cancer cells through its influence on activation of apoptotic signaling and inhibition of cell cycle progression. Piperine is known to affect cancer cells in variety of other ways such as influencing the redox homeostasis, inhibiting cancer stem cell (CSC) self-renewal and modulation of ER stress and autophagy. Piperine can modify activity of many enzymes and transcription factors to inhibit invasion, metastasis, and angiogenesis. Piperine is a potent inhibitor of p-glycoprotein (P-gp) and has a significant effect on the drug metabolizing enzyme (DME) system. Because of its inhibitory influence on P-gp activity, piperine can reverse multidrug resistance (MDR) in cancer cells and acts as bioavailability enhancer for many chemotherapeutic agents. In this article, we emphasize the potential of piperine as a promising cancer chemopreventive agent and the knowledge we collected in this review can be applied in the strategic design of future researches particularly human intervention trials with piperine.

## Introduction

Cancer is a deadly disease affecting human health in today's world and causes a huge economic and human loss throughout the world (Siegel et al., 2017). Several genetic, environmental, and lifestyle factors can contribute to a higher risk of cancer development in humans. Interestingly, only 5–10% of all cancer-related cases are due to genetic defects (mutations) inherited from a parent, whereas the remaining 90–95% of cancers are triggered by environmental and lifestyle factors (Anand et al., 2008). The lifestyle factors often associated with cancer development include unhealthy diet (such as processed foods, red meat), cigarette smoking, environmental carcinogens, ultraviolet (UV) exposure, stress, obesity, and physical inactivity (Anand et al., 2008; Parsa, 2012; Katzke et al., 2015). Diet alone contributes to ~30–35% of cancer-related demises (Doll and Peto, 1981). According to GLOBOCAN estimates, the global burden of new cancer incidences is projected to increase from 12.7 million in 2008 to 20.2 million by 2030 (Bray et al., 2012). Various dietary agents and “whole foods” have potent cancer preventive properties, but to date only a few of these dietary agents have shown efficacy in human intervention trials (Scott et al., 2009). Effectiveness of cancer prevention depends on the identification of potential risk factors and understanding how, at the molecular level, these factors trigger cancer initiation and progression (Sloan and Gelband, 2007). A better understanding of the signaling pathways and molecular events involved in cancer initiation can be acquired from following up patients with a higher risk for a specific type of cancer. Approximately 40–50% of cancers can be reduced if the existing knowledge about potential risk factors is taken into account by public health strategists (Stewart et al., 2016).

Many dietary components (owing to their potential carcinogenic and mutagenic properties) can initiate tumor growth in animal models and transform normal cells into mass of neoplastic cells (Zielinski, 2014). However, many epidemiological evidences have indicated that regular consumption of healthy diet in the form of fruits and vegetables can markedly reduce the risk of specific cancers (González-Vallinas et al., 2013). Phytochemicals, the non-nutritive components in the plant-derived diet, have recently garnered considerable attention for their role in prevention of cancer (Landis-Piwowar and Iyer, 2014). The World Health Organization (WHO) has laid down certain dietary guidelines to help people minimize the risk of acquiring a particular cancer type. Nutrigenomics can substantially refine our understanding of the complex interactions of phytochemicals with cancer cells and their potential role in prevention of cancer (Braicu et al., 2017). Nevertheless, cancer prevention by dietary phytochemicals is accepted as an inexpensive, readily applicable and accessible approach. Therefore, it is advisable to educate people regarding the consumption of phytochemicals as a cancer-preventive strategy (Pem and Jeewon, 2015).

Dietary phytochemicals have long been used for their potential as cancer preventive agents, which has lead to the development of cancer chemoprevention (Wattenberg, 1985). Many of these phytochemicals have entered into intervention trials for their potential role in cancer chemoprevention (Hosseini and Ghorbani, 2015). Piperine, a widely consumed dietary phytochemical, has shown cancer preventive properties both in cell cultures and animal models. In this review article, we discuss the molecular mechanisms underlying the chemopreventive action of piperine and their relevance to human health.

## Importance of piperine in food

**Figure 1:**
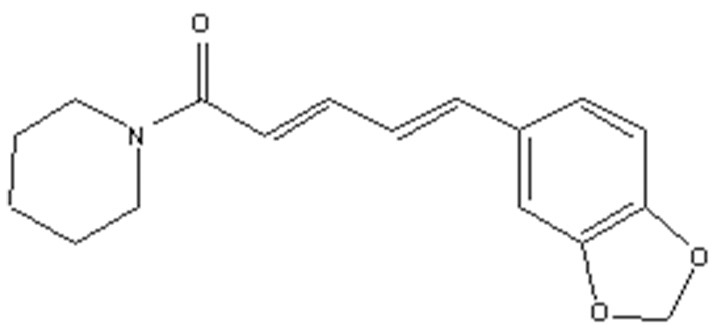
Structure of piperine. Piperine is an alkaloid containing a heterocyclic ring structure and a basic nitrogen atom. Nitrogen atom is located inside the ring structure.

Piperine (1-Piperoylpiperidine, Figure 1) is the most important dietary alkaloid predominantly found in the fruits and roots of *Piper nigrum* L. (black pepper) and *Piper longum* L. (long pepper) species of Piperaceae family (Zheng et al., 2016). Black pepper, often termed as the “king of spices,” has been exploited in Indian Systems of Medicine for the treatment of gastrointestinal and respiratory ailments (Gorgani et al., 2017). The characteristic pungency and biting taste of pepper is due high content of piperine in it. Piperine has been exploited for many therapeutic purposes in the past and is anticipated to remain so in the future. Piperine is an important dietary phytochemical due to its presence in spicy foods as well as its pharamacological activities (antiinflammatory, antimetastatic, anti-cancer, larvicidal, leishmanicidal, immunosuppressive, antimycobacterial, and antiparasitic activities) (Freire-de-Lima et al., 2008; Lu et al., 2012; Sahi et al., 2012; Rafiq et al., 2015; Rodgers et al., 2016; Samuel et al., 2016; Philipova et al., 2017; Soutar et al., 2017). Piperine was first separated from the extracts of pepper by Hans Christian Orsted in 1819 (Gorgani et al., 2017). Piperine, along with its isomers isopiperine, chavicine, and isochavicine, belongs to the family of alkaloids (Gorgani et al., 2017). Alkaloids constitute a group of natural occurring organic compounds that contain a ring structure and a basic nitrogen atom. By and large, the nitrogen atom is situated inside the heterocyclic ring structure (Cordell, 1981). Alkaloids have long been used as a reservoir for drug discovery infrastructure and, some of these alkaloids have already been approved by the US Food and Drug Administration (FDA), such as camptothecin, a famous inhibitor of topoisomerase I, and vinblastine, which interacts with tubulin leading to mitotic catastrophe (Lu et al., 2012). The cancer preventive impacts of piperine against a few sorts of cancer-causing agents, for example, 7,12-dimethyl benz(a)anthracene and benzo(a)pyrene, demonstrate its potential as a cancer chemopreventive agent (Selvendiran et al., 2004; Vellaichamy et al., 2009). To better understand the mechanistic role of piperine in cancer chemoprevention, it will be useful to know how cancer develops.

## Cancer development: an overview

**Figure 2:**
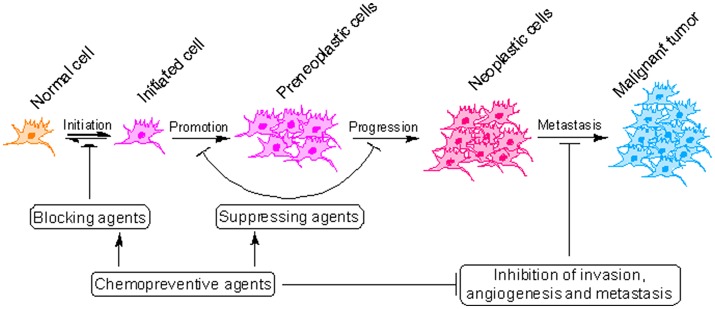
Critical steps in the process of cancer development. Cancer initiates with the transformation of a normal cell into an initiated cell, which undergoes tumor promotion to form preneoplastic cells and finally progress to neoplastic cells. Cancer chemopreventive agents can interfere with initiation (blocking agents) or later steps of this multi-stage process (suppressing agents).

Cancer development is a slow and dynamic process in which a genetically altered cell undergoes malignant transformation to form a mass of neoplastic cells (Cooper, 2000). Cancer development consists of atleast three separate but interconnected stages: initiation, promotion and progression (malignant conversion) where cancer can be interrupted using pharmacologically active agents (Figure 2) (Landis-Piwowar and Iyer, 2014). Although cancer is a difficult to treat disease, it can be prevented (Anand et al., 2008). As a matter of fact 30–40% of all cancer deaths can be prevented through appropriate dietary modifications and minimizing exposure to dietary and environmental carcinogens (Donaldson, 2004). Majority of cancer chemopreventive agents are present in fruits, vegetables and spices and can influence cancer cells in variety of ways (Rajesh et al., 2015; Baena Ruiz and Salinas Hernandez, 2016). However, validation of the mechanisms by which these components suppress cancer is obligatory before they can be prescribed for consideration in dietary servings or before they can be tried in human intervention trials (Greenwald, 2002). Piperine, a pharmacologically safe alkaloid, has been studied extensively for its chemopreventive properties (Gorgani et al., 2017). In this review, we will discuss the concept of cancer chemoprevention and analyzed the recent advances in chemopreventive action of piperine at the molecular, cellular and organism level which can be useful in design of future researches and exploration of new molecular targets for therapeutic intervention.

## Mechanism of cancer chemoprevention: an overview

**Figure 3:**
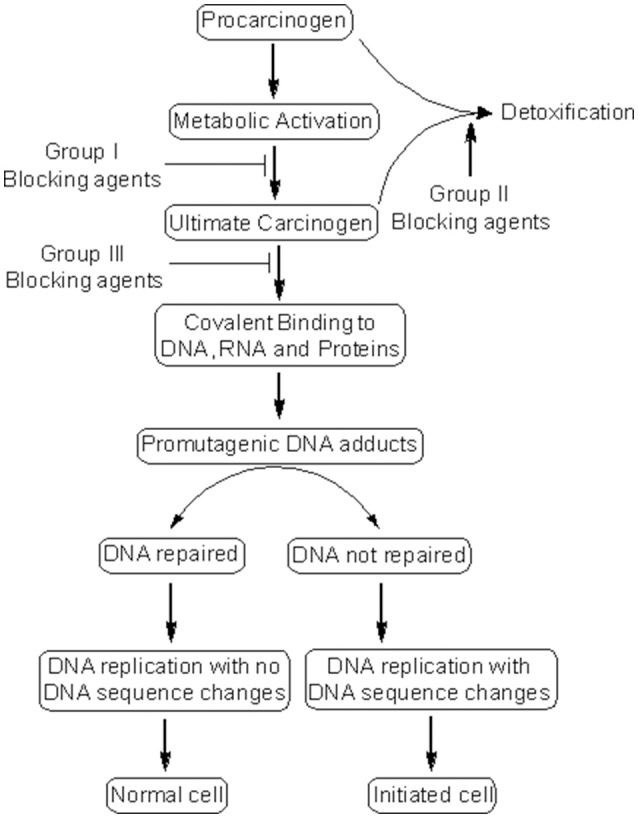
Mechanism of cancer chemoprevention. Cancer chemopreventive agents can inhibit the metabolic conversion of procarcinogens to their ultimate reactive intermediates (group I blocking agents) and their subsequent interaction with DNA, RNA, and proteins (group III blocking agents). Alternatively, blocking agents can stimulate the detoxification of carcinogens (group II blocking agents), leading to their removal from the body. Other chemopreventive agents (suppressing agents) can suppress the later steps (promotion, progression) of cancer development. Some chemopreventive agents can behave as both blocking and suppressing agents.

Cancer chemoprevention encourages the use of natural or synthetic agents to interrupt the process of cancer development by blocking or suppressing specific molecular events and signaling pathways associated with cancer development (Landis-Piwowar and Iyer, 2014). Chemopreventive agents have been divided into blocking agents and suppressing agents (Figure 2) (Wattenberg, 1985). Blocking agents are inhibitors of tumor initiation. Suppressing agents, on the other hand, suppress the transformation of initiated cells into preneoplastic and/or neoplastic cells (Wattenberg, 1985). Blocking agents are further sub-categorized into three groups according to their mechanism of action (Figure 3). Usually, combination chemopreventive strategy is preferred over single-agent chemoprevention. Combination chemopreventive approach utilizes multiple chemopreventive agents at low doses to achieve maximum chemopreventive efficacy with minimum toxicity (Chen and Malhotra, 2015). For example, GLAD, a cocktail of gefitinib, licofelone, atorvastatin, and α-difluoromethylornithine, suppresses colon tumorigenesis in APC(Min/+) mice with no toxicity (Mohammed et al., 2013). Combination chemoprevention strategies raise the possibility that whole foods might render “pharmacodynamic synergy” where the impact because of blend of numerous phytochemicals is more prominent than the additive sum of impacts of individual phytochemicals (Ullah and Ahmad, 2016).

Clinically, cancer chemoprevention has been classified as primary, secondary, and tertiary prevention. Primary chemoprevention is implemented to block the development of premalignant lesions, whereas secondary chemoprevention concentrates on suppressing the progression of these lesions to cancer, and tertiary chemoprevention aims to prevent the relapse or dissemination of a primary cancer (Greenwald, 2002; Steward and Brown, 2013). The ultimate goal of all forms of chemopreventive approaches is to decrease the rate of cancer incidence and cancer related deaths (Wattenberg, 1985). Nevertheless, there are several limitations that hamper the clinical use of chemopreventive agents such as the monetary costs and time required for directing epidemiological studies; separation and purification of these molecules; pharmacokinetic and pharmacodynamic properties of these agents (e.g., low bioavailability, low solubility, low stability, drug metabolism, etc.). Notably, piperine is profoundly hydrophobic and practically insoluble in water, which might hamper its use in clinics. Given that piperine is consumed widely as an integral part of human diet; piperine can be administered to human subjects with minimum toxicity and assessed for clinical efficacy.

## Chemopreventive mechanisms of piperine

The importance of cancer chemoprevention was recognized early in human history with identification and development of raloxifene and tamoxifen for breast cancer prevention and a series of agents that can cure cutaneous preneoplastic lesions (Kelloff et al., 1999; Wu et al., 2011). The first translational study of a potential chemopreventive agent was conducted using 13-cis retinoic acid (a derivative of vitamin A) which showed a significant size reduction in premalignant lesions of oral leukoplakia and prevented primary head-and-neck tumors (Lippman and Hong, 1992). The main chemopreventive mechanisms of action of piperine include activation of apoptotic signaling cascades, inhibition of cell proliferation, cell cycle arrest, alterations in redox homeostasis, modulation of ER stress and autophagy, inhibition of angiogenesis, induction of detoxification enzymes, and sensitization of tumors to radiotherapy and chemotherapy (Manayi et al., 2017). The aforementioned mechanisms of action of piperine reveal that piperine can contribute significantly to cancer chemoprevention.

### Piperine causes apoptosis in cancer cells, both *in Vitro* and *in Vivo*

**Figure 4:**
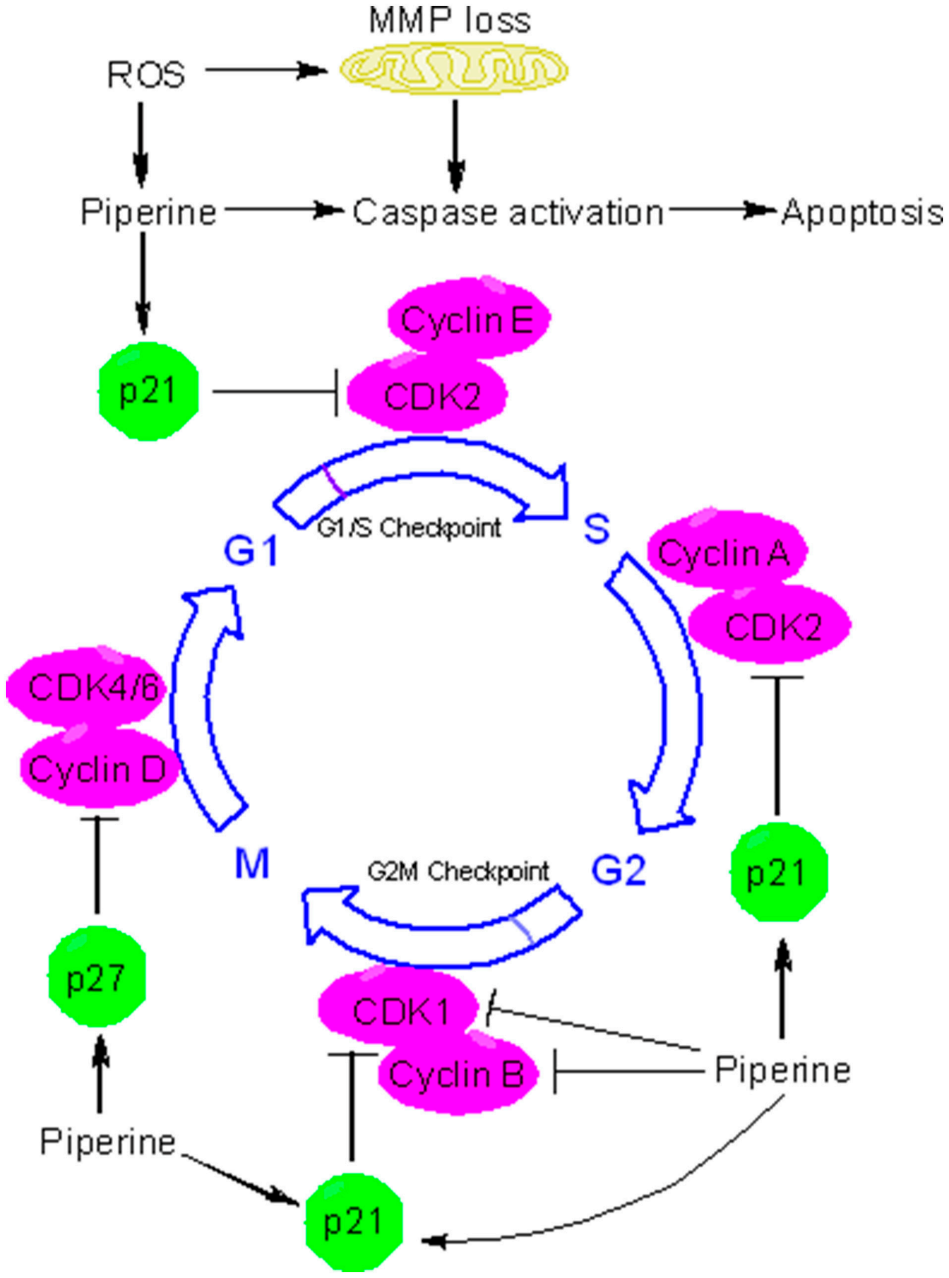
Piperine triggers apoptosis and causes cell cycle arrest. Piperine is a pro-oxidant agent and can stimulate the formation of reactive oxygen species (ROS) in many types of cancer cells. ROS triggers the depolarization of mitochondrial membrane potential (MMP), leading to release of cytochrome c, activation of caspases, and induction of apoptosis. Piperine can activate both intrinsic and extrinsic pathways of apoptosis. In addition to inducing caspases mediated apoptosis, piperine can inhibit cell proliferation via cell cycle arrest. Piperine can induce cell cycle arrest by directly binding to several target protein and depending on the cell type and behavior of tumor, it can arrest cell cycle at G1, G1/S, or at the G2/M phase. At the G1 phase, piperine halts cell cycle progression through downregulation of cyclin D and upregulation of p21. p21 is an inhibitor of cyclin dependent kinases (CDKs). In particular, p21 inhibits CDK2-cylcin E complex, with the consequent inhibition of CDK2-dependant phosphorylation of pRb and attenuation of E1F2, thus blocking transcription induced by E2F-1 and progression into and through S phase. P21 also inhibits the activity of CDK2-cyclin A and CDK1-cyclin B which are necessary for progression through S phase and G2/M respectively. Piperine upregulates the expression of p27. p27 can influence cell cycle in several ways. In particular, p27 blocks the activity of CDK4-cyclin D and CDK6-cyclin D, causing G1 cell cycle arrest.

Many chemopreventive agents are known to induce apoptosis and are used to retard tumor growth. Apoptosis is usually executed through two major pathways: mitochondria-mediated intrinsic pathway and death receptor-mediated extrinsic pathway (Tanaka, 2013). At the molecular level, piperine can influence many effector proteins engaged in apoptotic process and can activate both intrinsic and extrinsic pathways of apoptosis. Piperine suppressed the tumor development and metastasis in a mouse 4T1breast tumor model (Lai et al., 2012). Administration of piperine to 4T1 cells activated caspase 3-mediated intrinsic pathway of apoptosis and induced G2/M phase cell cycle arrest through attenuation of cyclin B1 expression (Lai et al., 2012)(Figure 4). Piperine treatment markedly decreased tumor growth in nude mice model xenografted with the androgen dependent (PC3) and androgen independent (LNCaP, DU145) prostate cancer cells (Samykutty et al., 2013). Piperine's inhibitory influence on growth of prostate cancer cells intercedes through decreased expression of phosphorylated STAT-3 and nuclear factor-κB (NF-κB) (Samykutty et al., 2013). Piperine treatment likewise impaired the expression of androgen receptor (AR) in LNCaP cells. Therefore, piperine can be utilized as a potential chemopreventive agent in the management of prostate cancer. Piperine is a potent inhibitor of cancer-associated angiogenesis and tumor invasiveness (Doucette et al., 2013). The invasiveness and metastatic behavior of cancer cells is often influenced by matrix metalloproteinases (MMPs) and specific expression of MMP-1, MMP-3, MMP-9, and MMP-13 has been related with metastasis and invasiveness of breast cancer cells *in vitro* (Balduyck et al., 2000). Piperine significantly reduced metastatic behavior of 4T1 cells by reducing the expression of MMP-9 and MMP-3 (Lai et al., 2012). Piperine suppresses phorbol-12-myristate-13-acetate (PMA)-induced MMP-9 expression through its inhibitory influence on protein kinase C-α (PKCα)/extracellular signal-regulated kinase (ERK) 1/2 activity and inhibition of NF-κB/AP-1 activity (Hwang et al., 2011). These studies indicate that piperine might have a role in modulating signaling networks associated with epithelial-to-mesenchymal transition (EMT), a process that regulates metastasis, cancer stem cell (CSC) self-renewal and intratumoural heterogeneity (Chaffer et al., 2016; Kim et al., 2017; Shibue and Weinberg, 2017). Piperine is also a very good antimetastatic agent against lung carcinogenesis initiated by B16F10 mouse melanoma cells in mice (Pradeep and Kuttan, 2002) and stifled PMA-induced invasiveness of human fibrosarcoma HT-1080 cells (Hwang et al., 2011). Oral supplementation of piperine markedly reduced the DNA damage and DNA-protein crosslinks in experimental model of benzo(a)pyrene induced lung carcinogenesis (Selvendiran et al., 2006). Piperine appears to extend its chemopreventive effects against lung carcinogenesis through the modulation of serum and tissue glycoprotein levels, which are one of the key biomarkers of neoplastic transformation (Selvendiran et al., 2006). Piperine displays excellent antitumor efficacy against human HER2-overexpressing breast cancer cells through its inhibitory influence on ERK1/2 signaling and blockade of SREBP-1 and FAS expression (Do et al., 2013). Piperine is a potent inhibitor of epidermal growth factor (EGF)-induced MMP-9 expression and acts through inhibition of NF-κB and AP-1 activation and by intervening with ERK1/2, p38 MAPK, and Akt signaling pathways, resulting inhibition of cell migration. Therefore, piperine can serve as a promising chemopreventive agent for human breast cancer with HER2 overexpression (Do et al., 2013). Many regulators of cell survival are such as NF-κB, c-Fos, CREB, and ATF2 are strongly inhibited by piperine (Pradeep and Kuttan, 2004). Piperine is also an inhibitor of survivin and this information has been exploited for therapeutic intervention of neuroblastoma, an embryonically derived tumor (Muthukumar and Vanisree, 2011; Sattarinezhad et al., 2015). Survivin is an important anti-apoptotic protein that is significantly up-regulated in neuroblastoma (Hagenbuchner et al., 2016). Piperine can inhibit the activity of enzyme EGFR tyrosine kinase, which is one of the key targets of potential chemopreventive agents (Kelloff et al., 1996; Paarakh et al., 2015). Recent evidences have shown that piperine can be used to suppress cancer development by targeting human G-quadruplex DNA sequences (Tawani et al., 2016). G-quadruplex DNA structures are four stranded DNA structures that are generated by square planar arrangement of G-quartets during DNA metabolism and play vital role in regulation of cellular processes that might contribute to cancer development. Piperine binds with high affinity to G-quadruplex DNA and in particular to G-quadruplex structure formed at c-myc promoter region (Tawani et al., 2016). The ability of piperine to bind G-quadruplexe structures makes it useful as a potent chemopreventive agent for cancers with aberrations in DNA metabolism.

### Piperine alters the redox homeostasis in a cell and tissue specific way to retard cancer development

Reactive oxygen species (ROS), free radicals, and ultimate reactive forms produced from metabolic activation of procarcinogens are known to assume a critical role in cancer development (Liou and Storz, 2010). Both enzymatic and non-enzymatic reactions contribute to ROS formation (Di Meo et al., 2016). The enzymatic sources that create ROS include a series of enzymes such as NADPH oxidase, inducible nitric oxide synthase (iNOS), xanthine oxidase (XO), lipoxygenase, cyclooxygenase, and cytochrome P450 enzyme system (Bachi et al., 2013). ROS are also generated from non-enzymatic activity of electron transport chain in mitochondria (Murphy, 2009). Piperine mediated redox changes can influence cellular physiology in variety of ways which may be cell or tissue specific as well as dose dependent. So, depending on the context, piperine can enhance cell survival or commit the cell to death. Piperine has been demonstrated to impart protection against oxidative stress-mediated cellular damage by quenching ROS, free radicals and reactive metabolic intermediates (Mittal and Gupta, 2000; Srinivasan, 2007). Piperine is a potent antioxidant particularly at low concentrations and can potentially mitigate ROS mediated oxidative stress (Mittal and Gupta, 2000). Therefore, piperine can suppress oxidative stress and delay cancer development (Damanhouri and Ahmad, 2014). Piperine has been proven to lessen the oxidative modifications induced by chemical carcinogens (7,12-dimethyl benzanthracene, dimethyl aminomethyl azobenzene, and 3-methyl cholanthrene) in a rat model of colon cancer (Khajuria et al., 1998). It was observed that these carcinogens depleted glutathione and substantially enhanced formation of thiobarbituric reactive substances (TBARS). However, administration of piperine inhibited TBARS formation, increased the glutathione levels and restored γ -GT and Na+, K+-ATPase activity, accounting for the protective role of piperine against carcinogen induced oxidative damage (Khajuria et al., 1998). Oral supplementation of piperine in mice model of lung cancer decreased oxidative stress mediated mitochondrial lipid peroxidation and enhanced the activities of both enzymatic (superoxide dismutase, catalase, and glutathione peroxidase) and non-enzymatic (reduced glutathione, vitamin E, and vitamin C) anti-oxidant defense system (Selvendiran et al., 2006).

Apart from its antioxidant effects, piperine possesses potent pro-oxidant activity and has long been known to affect the redox state of cancer cells (Martin-Cordero et al., 2012). Because of profound alterations in their metabolism and signaling pathways, cancer cells generate high levels of ROS that ends up in a state of increased basal oxidative stress (Liou and Storz, 2010). This state of increased oxidative stress makes cancer extremely susceptible to pro-oxidant agents that increase the formation of ROS to a level where they become cytotoxic. Piperine, usually at higher concentrations, acts as a potent prooxidant agent, resulting in increased generation of free radicals. For example, piperine activates radical-mediated mitochondrial pathway of apoptosis in hepatocellular carcinoma cells (Figure 4) (Gunasekaran et al., 2017). In the recent times, redox mediated cancer therapeutics have garnered much interest due to their anticancer activity (Wondrak, 2009; Gunasekaran et al., 2017). Piperine induced ROS generation and subsequent cell death has been studied in many types of cancer cells (Figure 4) (Yaffe et al., 2013). Administration of piperine-loaded nanofiber mats to HeLa and MCF7 cancer cells caused a substantial ROS generation leading to cell death (Jain et al., 2016). Piperine stimulates the generation of ROS in human oral squamous cells which in turn leads to dissipation of mitochondrial membrane potential (MMP), activation of caspases, and cell cycle arrest (Figure 4) (Siddiqui et al., 2017). Piperine also causes ROS-dependant cell death and cell cycle arrest in HRT-18 rectal adenocarcinoma cancer cells (Yaffe et al., 2013). The ability of piperine to induce oxidative stress-mediated apoptosis in cancer cells makes it a potential chemopreventive agent specific for cancer cells.

### Piperine causes cell cycle arrest

The cell cycle is fundamental to maintain continuity in cell proliferation and to ensure the protection of proliferating cells from DNA damage. The fundamental protein regulators of cell cycle are cyclin-dependent kinases (CDKs), cyclins, and CDK inhibitors (CKIs). Cancer development is often associated with loss of cell cycle regulation (Collins et al., 1997; Feitelson et al., 2015). Many chemopreventive agents are known to modulate cell cycle progression as a part of their chemopreventive mechanism (Meeran and Katiyar, 2008). Piperine has been implicated in arresting cancer cells at different phases of cell cycle progression via induction and inhibition of different protein regulators and checkpoints (Figure 4). For example, piperine arrests SKMEL-28 and mouse B16F0 melanoma cells at G1 phase through the downregulation of cyclin D1 and induction of p21(Fofaria et al., 2014). Piperine probably acts by inducing ROS mediated DNA damage which is indicated by the phosphorylation of H2AX at Ser139. As a result of piperine induced DNA damage, ataxia telangiectasia and rad3-related protein (ATR) and checkpoint kinase 1 (Chk1) are activated leading to cell cycle arrest and subsequent apoptosis (Fofaria et al., 2014). Piperine has long been held responsible for stimulating ROS formation in cancer cells. ROS blocking agents such as tiron have been found to protect cancer cells from piperine mediated cell cycle arrest and apoptosis (Fofaria et al., 2014). Piperine-induced ROS generation has been associated with apoptosis in B16F10 melanoma cells and HRT-18 human rectal adenocarcinoma cancer cells (Yaffe et al., 2013). Piperine arrests HT-29 colon carcinoma cell at G1 phase through the downregulation of cyclins D1 and D3 and their activating partners CDK4 and CDK6 as well as through the inhibition of phosphorylation of the retinoblastoma protein (pRb) and induction of p21 and p27 (Yaffe et al., 2015). Piperine has been found to arrest prostate cancer cells (including both androgen dependent, PC3 and androgen independent, LNCaP and DU145 cells) at G0/G1 phase via downregulation of cyclins (cyclin D1 and cyclin A) and upregulation of CDK inhibitors (p21 and p27). However, cell cycle arrest was less robust in PC3 than LNCaP and DU145 cells because of less noticeable p21 and p27 induction in PC3 (Ouyang et al., 2013). Apart from G0/G1 arrest, piperine arrests osteosarcoma cells at G2/M phase of cell cycle through the downregulation of cyclin B1 and enhanced phosphorylation of cyclin-dependent kinase-1 (CDK1) and checkpoint kinase 2 (Chk2) (Zhang et al., 2015). Piperine induced G2/M phase cell cycle arrest can has also be noticed in HeLa and MCF-7 cells, and in this case piperine-induced ROS generation is held responsible for the induction of cell cycle arrest (Jain et al., 2016). Piperine can be useful in chemoprevention of TNBC and hormone-dependant breast cancer cells and without influencing normal mammary epithelial cell growth. Piperine blocks growth and invasiveness of TNBC cells through the downregulation of G1-associated (cyclin D3, CDK4, E2F-1) and G2-associated (cyclin B, CDK1, Cdc25C) proteins, as well as an induction of p21 (Greenshields et al., 2015). Notably, it is the activation of caspases and not ROS formation that participates in piperine-induced cell death in TNBC cells. The growth inhibitory influences of piperine on cell cycle progression suggest that piperine may emerge as promising cancer chemopreventive agent.

### Piperine inhibits the cancer stem cells (CSCs) self-renewal

**Figure 5:**
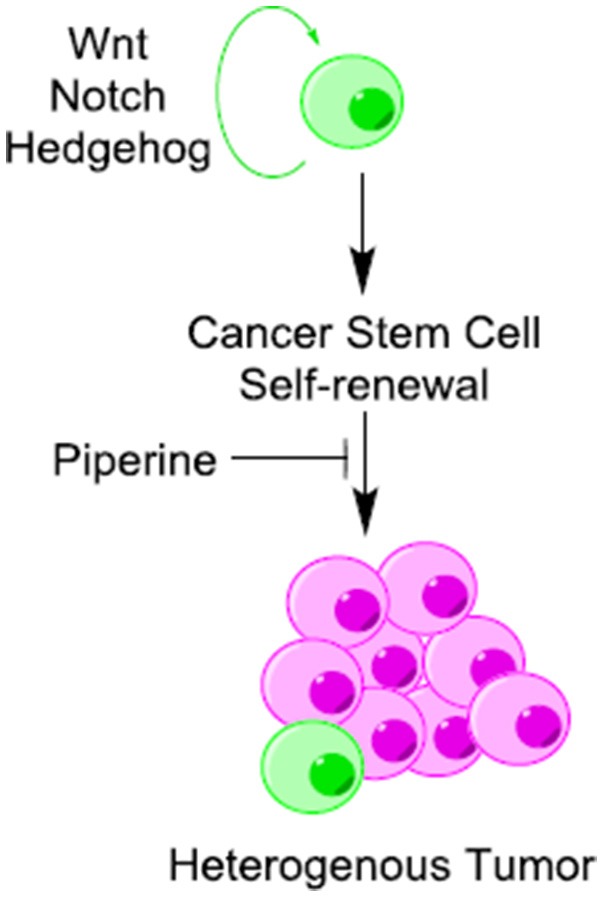
Piperine is a potential inhibitor of cancer stem cells (CSCs) self-renewal. Piperine inhibits cancer stem cells (CSCs) self-renewal through its direct or indirect inhibitory influence on Wnt/β-catenin, Hedgehog and Notch pathways.

Cancer stem cells (CSCs) are known to exist in several forms of human cancers and have profound implications for cancer chemoprevention. Cancer stem cells undergo continuous self-renewal and differentiation, giving rise to a heterogeneous mass of cancer cells (Li et al., 2011). Aberrant self-renewal of CSC contributes to tumor initiation and relapse of cancers (Economopoulou et al., 2012). Wnt/β-catenin, Hedgehog, and Notch are three fundamental signaling pathways that regulate self-renewal and differentiation in CSCs (Figure 5) (Wang et al., 2016). All of these pathways are influenced directly or indirectly by piperine. Piperine inhibits Wnt/β-catenin signaling pathway in breast CSCs (Kakarala et al., 2010) and modifies the self-renewal properties of CSCs (Kim et al., 2012). Piperine plays an important role in maintaining a balance between dividing and quiescent cells by influencing key regulating proteins in the signaling network such as DKK-1, secreted frizzled-related protein 2 (sFRP2), B cell-specific Moloney murine leukemia virus integration site 1 (Bmi-1) and cyclin-dependent kinase 6 (CDK6) (Kim et al., 2012). Piperine has recently been found to inhibit the mammosphere formation of breast cancer cells (Economopoulou et al., 2012). However, it is not clear whether the inhibitory effect of piperine on mammosphere formation is due to direct interaction of piperine with Notch or via induction of alterations in Wnt signaling. Piperine has also been effect hedgehog signaling through its influence on nuclear import and activation of NF-κB, although the effect on hedgehog signaling is not clear.

### Piperine influences autophagy and ER stress in favor of cell death

Piperine inhibits the growth and proliferation of prostate cancer cells through induction of autophagy as indicated by increase in LC3II level in LNCaP and PC3 cells (Ouyang et al., 2013). This can be confirmed by a cotreatment of piperine with lysosomal inhibitor chloroquine that leads to an even enhanced formation of LC3B puncta in LNCaP and PC3 cell level, thus indicating that piperine induces autophagy flux (Ouyang et al., 2013). Piperine is effective inhibitor of mTOR, a key negative regulator of autophagy. Piperine exhibits mTORC1 inhibitory activity in Caco-2 and HT-29 cells (Moreau and Kaur, 2017). Piperine blocks the growth of HT-29 colon cancer cells through induction of autophagy and induction of pro-apoptotic components of ER stress such as CHOP, GRP78, IRE1α, and JNK and inhibition of Akt phosphorylation and survivin expression. CHOP, also known as GADD153, is a member of C/EBP family of transcription factor and plays a vital role in endoplasmic reticulum (ER) stress-mediated apoptosis (Yaffe et al., 2015).

### Piperine inhibits angiogenesis

Angiogenesis, the phenomenon of formation of new blood vasculature from preexisting vasculature, plays an important role in the late stages of cancer development, allowing tumors to grow in size and metastasize (Sharma et al., 2001). Piperine influences many aspects of angiogenesis. Recently piperine has been shown to inhibit proliferation, migration, and tubule formation by human umbilical vein endothelial cells (HUVECs) (Karar and Maity, 2011; Doucette et al., 2013). HUVECs are often used as a model system to understand the regulation and development of angiogenesis. Piperine suppressed collagen-induced angiogenesis in rat aorta ring explant cultures and breast cancer cell-induced angiogenic activity in chick embryos. At the molecular level, piperine blocks Akt phosphorylation at Ser 473 and Thr 308 residues, leading to inhibition of phosphoinositide-3 kinase (PI3K)/Akt signaling which is a key regulator of angiogenesis and endothelial cell function (Doucette et al., 2013). Vascular endothelial growth factor (VEGF) is potent activator of PI3K/Akt signaling in endothelial cells (Abid et al., 2004). Therefore, when endothelial cells are stimulated by VEGF, activation of the PI3K pathway within these cells triggers cell migration (Karar and Maity, 2011). Piperine blocks angiogenesis in breast carcinoma syngraft through its inhibitory influence on VEGF expression (Talib, 2017). Piperine in the form of *P. longum*, has also been found to inhibit VEGF and proinflammatory cytokines and B16F10 melanoma cell-induced angiogenesis in C57BL/6 mice (Sunila and Kuttan, 2006). VEGF is also an important component of tumor microenvironment. Therefore, angiogenesis is directly influenced by tumor microenvironment. The tumor microenvironment consists of mixture of extracellular matrix (ECM) molecules, tumor cells, endothelial cells, cancer associated fibroblasts and immune sensitive cells. Piperine has been reported to influence tumor microenvironment raising the possibility of its use in the treatment and/or prevention of cancer (Casey et al., 2015).

### Piperine acts as an adjuvant for TRAIL based therapy of TNBC

Human triple negative breast cancer (characterized by the deficiency of estrogen receptor, progesterone receptor, and HER2) is the most dangerous type of human breast cancer (Andergassen et al., 2017). Tumor necrosis factor-related apoptosis-inducing ligand (TRAIL) is often used to induce apoptosis selectively in cancer cells (Mérino et al., 2007). However, the clinical use of TRAIL-based therapy is often limited due to resistance induction. Several methods to overcome TRAIL resistance of cancer cells have been proposed, including combination of TRAIL with dietary phytochemicals. Piperine is a potent adjuvant of TRAIL-mediated cell death and promotes TRAIL induced cell death in both TRAIL-sensitive and TRAIL-resistant triple-negative breast cancer cells through the inhibition of survivin and p65 phosphorylation (Abdelhamed et al., 2014).

### Piperine can influence the activity of drug metabolizing enzyme system

Piperine possesses chemopreventive effects by blocking the metabolic activation of certain pro-carcinogens by the drug metabolizing enzyme (DME) system (Reen et al., 1996). Piperine inhibits the major drug-metabolizing enzyme CYP3A4 (Bhardwaj et al., 2002) and many other DMEs which include arylhydrocarbon hydroxylase (AHH), uridine diphosphate-glucoronyl transferase (UDP-GT), UDP-glucose dehydrogenase (UDP-GDH), 5-lipooxygenase, cyclooxygenase-1, and cytochrome P450 (Atal et al., 1985). It is generally believed that stimulation of glutathione-metabolizing enzymes (e.g., GPx, GR, and G6PDH) offers protective advantage to cells against various reactive metabolites such as ROS and ultimate reactive forms of carcinogens (Selvendiran et al., 2005). Piperine supplementation in mice model of lung carcinogenesis markedly reduced the activity of phase-I enzymes (NADPH-C reductase, cyt-p450, and cyt-b5) but caused a substantial increase in glutathione-metabolizing enzymes (GPx, GR, and G6PDH), accounting for its role in cancer chemoprevention (Selvendiran et al., 2005).

### Piperine inhibits the P-glycoprotein activity

Development of multidrug resistance (MDR) in cancer is a severe problem that limits the use of chemotherapeutics in successful treatment of cancer (Gottesman and Pastan, 2015). Highly invasive phenotype associated with malignant cancers is often due to over expression of p-glycoprotein (P-gp) (also known as MDR1 or ABCB1). P-gp, a 170 kDa membrane linked protein, belongs to the ABC (ATP-binding cassette) superfamily and imparts resistance to cancer cells by acting as an ATP- dependent efflux pump for an incredible series of chemotherapeutic agents (Ambudkar et al., 2003). Piperine is potent inhibitor of P-gp and MRP-1(Manayi et al., 2017). Piperine binds between the consensus sequence of Walker A/P loop and Walker C loop (linker peptide) at the nucleotide binding domain which is crucial for ATP coupled efflux through P-gp (Singh et al., 2013). Piperine competes with ATP binding site in P-gp and with this mind recently two low molecular weight piperine analogs Pip1 and Pip2 were synthesized which show better interaction with P-gp than piperine *in silico* (Syed et al., 2017). Both of these analogs when co-administered with certain drugs (such as vincristine, colchicine or paclitaxel) could reverse drug resistance in P-gp overexpressing KB (cervical) and SW480 (colon) cancer cells.

### Piperine enhances the bioavailability of drugs

Because of its inhibitory influence on P-gp activity, piperine acts an efficient bioavailability enhancer for a series of chemotherapeutic agents (Bhardwaj et al., 2002). Piperine is regarded as the world's first scientifically validated bioavailability enhancer (Atal and Bedi, 2010). Different mechanisms have been postulated to explain the bioenhancer activity of piperine. For example, piperine inhibits P-gp and cytochrome P450 3A4 (CYP3A4), both of which contribute to first-pass elimination of many drugs (Atal and Bedi, 2010). CYP3A4 alone metabolizes ~50% of marketed drugs (Zhou, 2008). Other DMEs influenced by piperine include CYP1A1, CYP1B1, CYP1B2, CYP2E1, CYP3A4, etc. Therefore, all the drugs that are metabolized by these enzymes are affected by the administration of piperine, making piperine a non-specific inhibitor of different forms of cytochrome P-450 enzyme system (Atal et al., 1985). Piperine is an inhibitor of UDP-glucuronosyltransferase (Singh et al., 1986). Piperine substantially enhanced the bioavailability of many chemopreventive agents such as resveratrol mostly through the inhibition of glucuronidation. Therefore, when resveratrol is administered along with piperine, the plasma concentration of resveratrol is significantly enhanced (Johnson et al., 2011).

### Piperine enhances the radiosensitization of, otherwise resistant, tumors

Ionizing radiation (IR) has long been used in cancer therapy (Baskar et al., 2014). The purpose of using IR in cancer treatment is to ensure that cancer cells outright, or at minimum, become incapable of proliferation. However, dose-limiting normal tissue toxicity and radioresistance of certain tumors limit the clinical use of IR in cancer therapeutics (Kim et al., 2015). Many phytochemicals have been identified which can specifically lower the death threshold of cancer cells when used in combination with ionizing radiation. Such agents are known as radiosensitizers. Recent study has shown that piperine can enhance the radiosensitivity of cancer cells without influencing the sensitivity of normal cells (Tak et al., 2012). Piperine has been found to enhance IR-induced apoptosis in cancer cells presumably through increased ROS formation which leads to dissipation of MMP and subsequent cell death (Tak et al., 2012). Piperine has also been reported to act as potent ultraviolet (UV)-B photosensitizer in B16F10 mouse melanoma cells (Rafiq et al., 2015). Therefore, when used in combination with UVB, piperine elevates intracellular ROS formation and impairs intracellular calcium homeostasis which together with the attenuation of survival signaling pathways leads to enhanced cell death in B16F10 cells.

### Piperine influences UV-(B) signaling and induces apoptosis in UV irradiated skin cancer cells

Ultraviolet radiation (UV)-R causes DNA damage (mutations) and induces excessive generation of reactive oxygen species (ROS) which leads to cancer initiation and promotion. Piperine is a potential photochemopreventive agent. Piperine attenuates the (UV)-R induced DNA damage in human HaCaT keratinocytes through its influence on the NF-κB and Bax/Bcl-2 pathways (Verma et al., 2017). It is believed that piperine binds to active site of NF-κB and blocks its nuclear localization. Altered NF-κB activity in turn triggers suppression of apoptotic marker genes and induction of proteins involved in photoprotection (Verma et al., 2017). Piperine has been shown to elevate ROS formation and impair calcium homeostasis in B16F10 melanoma cells. Piperine acts as a potent UV photosensitizer for melanoma cells, causing cell death, and attenuation of major regulators of survival signaling pathways (Rafiq et al., 2015). It is believed that combination of piperine and UV may offer a possible and practical therapeutic strategy for melanoma in future.

## Future perspectives

There are a large number of alkaloids that can prevent the development of cancer. Many natural alkaloids have been screened, identified, and developed successfully into FDA-approved anticancer drugs. Chemoprevention, using phytochemicals from fruits, vegetables, and spices, is an important prophylactic strategy against cancer development. Piperine has recently garnered much attention for its chemopreventive properties. Piperine possesses potential chemopreventive properties which may be due to induction cell cycle arrest, increased cell apoptosis, disruption of redox homeostasis, attenuation of CSC self-renewal, inhibition of angiogenesis, modulation of ER stress, and autophagy, influence on the Wnt/β-catenin and inhibition of PI-3K/Akt signaling pathways. Notably, the hydrophobic nature and poor aqueous solubility of piperine limits the clinical use of this natural molecule. The information collected by us in this review has revealed that piperine can target different types of cancer cells specifically and manifest specific mechanisms of action in accordance with the type of cancer it acts on. However, the exact mechanism involved in the chemopreventive effects of piperine against the cancer development is not fully understood. Therefore, the precise mechanism of action of piperine must be validated before final recommendations on the clinical use of this natural molecule can be made. The hope is that piperine could be administered with minimum risk to human subjects and that it would produce a significant chemopreventive effect.

## Author contributions

RR conceived the idea and wrote the manuscript, MB edited the manuscript and helped to improve the quality of this review paper.

### Conflict of interest statement

The authors declare that the research was conducted in the absence of any commercial or financial relationships that could be construed as a potential conflict of interest.

## Abbreviations

AKT: protein kinase B
AP-1: activated protein-1
ATF2: activated transcription factor 2
ATR: ataxia telangiectasia and rad3-related protein
Bmi-1: B cell-specific Moloney murine leukemia virus integration site 1
CDK6: cyclin-dependent kinase 6
CDKs: cyclin dependant kinases
CHOP: C/EBP homologous protein
CYP3A4: cytochrome P450 3A4
Chk1: checkpoint kinase 1
CREB: cAMP response element-binding
DKK-1: dickkopf 1
EGF: epidermal growth factor
EGFR: epidermal growth factor receptor
FAS: fatty acid synthase
GADD 153: growth arrest and DNA damage-inducible gene 153
GRP78: glucose-regulated protein 78
HER2: human epidermal growth factor receptor 2
H2AX: H2A Histone Family Member X
IR: ionizing radiation
MAPKs: mitogen-activated protein kinases
MMP-2/-9: matrix metalloproteinase (MMP)-2/-9
NF-κB: nuclear factor kappa B
PARP: poly-ADPribose polymerase
PKCα: protein kinase C-α
ROS: reactive oxygen species
SREBP-1: sterol regulatory element-binding protein-1
TNBC: triple negative breast cancer
TRAIL: tumor necrosis factor-related apoptosis-inducing ligand
VEGF: vascular endothelial growth factor
sFRP2: secreted frizzled-related protein 2.
